# The Linkage Between Leadership Styles, Employee Loyalty, and Turnover Intention in Healthcare Industry

**DOI:** 10.3389/fpsyg.2022.890366

**Published:** 2022-04-28

**Authors:** Mochamad Vrans Romi, Nada Alsubki, Hana Mohammed Almadhi, Arfendo Propheto

**Affiliations:** ^1^Management Department, Jenderal Achmad Yani University, Cimahi, Indonesia; ^2^Department of Health Sciences, College of Applied Studies and Community Service, King Saud University, Riyadh, Saudi Arabia; ^3^International Business Management, Binus Business School, Bina Nusantara University, Jakarta, Indonesia

**Keywords:** leadership style, employee loyalty, turnover intention, healthcare, COVID-19

## Introduction

The coronavirus disease-2019 (COVID-19) pandemic in Indonesia has resulted in a heavy burden upon the country’s healthcare system, especially frontline health workers who are at constant risk of exposure to COVID-19 (Fahlevi and Alharbi, 2021a). Nearly two thousand health workers have died during the COVID-19 outbreak (BBC, 2021), and the death rate for health workers in Indonesia is the highest in Asia and the third-largest in the world. Apart from the risk of infection, another serious risk is the effect on workers’ mental health, including burnout syndrome and mental fatigue (Vizheh et al., 2020). Health workers traditionally have high exposure to very tremendous levels of stress, but no rules or policies currently exist that adequately protect them. Research conducted by a research team from the Master of Occupational Medicine Study Program, Faculty of Medicine, University of Indonesia (2020) determined that as many as 83% of health workers in Indonesia have experienced moderate and severe burnout syndrome, a statistic which threatens the effective operation of the healthcare system as a whole.

The high risk of healthcare workers suffering from burnout syndrome due to increased exposure to unusually severe stress during the pandemic can have long-term effects on the quality of medical services. Workers can feel depressed, experience extreme fatigue, and feel a lack of competence in carrying out their duties, all of which have a negative impact on efforts to fight COVID-19 (Chirico et al., 2020). The Research Team from the Master of Occupational Medicine Study Program, consisting of Dr. Ray W Basrowi, MKK; Dr. Levina Chandra Khoe, MPH; and Dr. Marsen Isbayuputra, SpOK, found that in addition to the percentage of workers experiencing burnout syndrome (Faculty of Medicine, University of Indonesia, 2020):

• Forty-one percent of health workers experienced moderate and severe emotional exhaustion, 22% experienced moderate and severe loss of empathy, and 52% experienced moderate and severe lack of confidence;
• Doctors who treated patients with COVID-19, both general practitioners, and specialists, were twice as likely to experience emotional exhaustion and loss of empathy as those who had not treated patients with COVID-19;
• Midwives who treated patients with COVID-19 were 2 times more likely to experience emotional exhaustion than those who had not treated patients with COVID-19;
• There were still health workers (2%) who had not received personal protective equipment (PPE) from their health facilities; and

• Approximately 75% of health facilities did not carry out routine swab checks and 59% did not carry out routine rapid tests for their health workers.

The Dean of the Faculty of Medicine, University of Indonesia (2020), Prof. Dr. Ari Fahrial Syam, SpPD-KGEH, MMB, stated that the research was a contribution from the University of Indonesia to the government to help identify the potential physical and mental health risks for medical personnel in Indonesia during a pandemic. According to Prof Ari, “various findings from this study can be a source of recommendations for protection intervention strategies and improving the health quality of Indonesian medical personnel, so that they are maximal in carrying out their medical service duties but also remain healthy.” The results of the study recommend that in addition to physical health and safety protection, hospital management, health facilities, and the government should begin prioritizing mental health interventions, such as psychological assistance and counseling for health workers, especially those whose work was directly impacted by the pandemic (Fahlevi and Alharbi, 2021b).

Another suggestion was the need to create a safe atmosphere for health workers, in which to carry out their duties by applying principles of comprehensive occupational medicine. The healthcare industry in Indonesia has long faced challenges in retaining employees. This high employee turnover rate disrupts optimal performance and the facilitation of healthcare services in the country (Kaye et al., 2021). The issue presents a serious problem in the economic development of Indonesia, already slowed by the pandemic (Purwanto A. et al., 2020). A high employee turnover rate can be equated with the style of leadership implemented in an organization (Supriadi et al., 2020), and, therefore, this research explores the impact of leadership style on employee loyalty and turnover in the healthcare industry.

## Methods

Saunders et al. (2009) defined the five major types of research philosophy: positivism, interpretivism, pragmatism, post-modernism, and critical realism. Each research philosophy has its ontology, epistemology, axiology, and typical methods. For this research, the philosophy was positivism, due to the implementation of a theory elaboration approach; an approach that is typically deductive and uses a qualitative research method. Sekaran and Bougie (2016) classified positivism researchers as natural scientists as they seek results by elaborating upon an existing conceptual framework (Purwanto H. et al., 2020).

This research implemented the theory elaboration approach for its methodology. Theory elaboration is “the process of executing and conceptualizing empirical research using a preliminary model and pre-existing conceptual framework as a foundation to develop new theoretical insights or to advance a theory by structuring, specifying, and contrasting” in a qualitative manner (Baxter and Jack, 2008). In this study, the previously developed conceptual framework has been elaborated upon to create a foundational structure. Previous research on leadership styles, employee loyalty, and turnover intention was deemed to be insufficient, as there is no focus on how leadership style impacts employee loyalty and intention, but rather, only which leadership style is the most suitable in enhancing these (Sunarsi et al., 2020). The study interviewed employees at X hospital, one of the largest private hospitals in Indonesia.

## Results and Discussion

Three coding mappings were generated from the data collected for this research, based on verbatim transcripts from semi-structured interviews of thirteen audio recordings: twelve employees and one human resource strategy senior manager. Firstly, three categories were generated from the audio recordings in Coding Mapping One, Leadership in Healthcare Industry: “Leadership Styles,” “The Leader-Member Exchange Relationships,” and “the most preferred leadership style to enhance employee loyalty and reduce turnover intention.”

## Coding Mapping One: Leadership in Healthcare Industry

**FIGURE 1:**
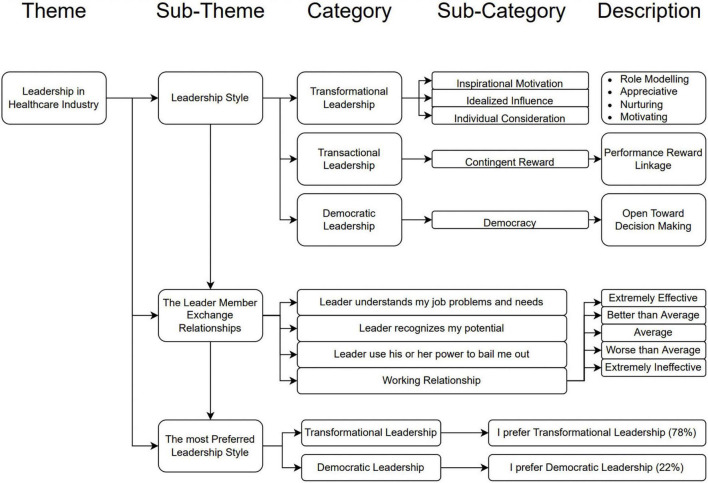
Coding mapping leadership styles in healthcare industry.

Coding Mapping One was used to interpreting the interviews, and investigate the leadership styles implemented in X hospital, leader-member exchange relationship, and the leadership style preferred by employees. Based on the coding mapping, it was discovered that X hospital implements three of five leadership styles, identified from the three categories: transformational leadership, transactional leadership, and democratic leadership. Figure 1 specifically describes the basic important factors of leadership style in healthcare industry.

## Transformational Leadership

Based on analysis of the interview transcripts, three sub-categories were identified from transformational leadership in X hospital: inspirational motivation, idealized influence, and individualized consideration.

### Inspirational Motivation

Inspirational Motivation is described as leadership that is motivating; with leaders who motivate their subordinates by being optimistic about the future, their work, and work progress rather than pessimistic which can lead to feelings of demotivation.

#### Employees’ Statements

##### Motivating

“My leader motivates me in a way that he discusses with us to find the solution in order to tackle the problem, and he is optimistic that the problem could be solved by implementing the solution.”

“My leader is very optimistic with our targets. He drives his team to believe that we are going to reach our targets and it creates motivation for us.”

### Idealized Influence

Idealized influence can be described as unselfish and role-modeling. Idealized Influence is an attribute of a transformational leader; he/she tends to be unselfish as he/she goes beyond self-interest. Leaders are looked up to as role model by their subordinates, gaining their respect and trust.

#### Employees’ Statements

##### Role-Modeling

“I look up to my leader. She gained my respect and trust because she uses her position to nurture us rather than to use it as leverage. I look up to her in the way she treats her subordinates well, in my opinion, she is a great leader.”

“I respect and trust my leader. I really admire and look up to him as he knows when to say “No” and “Yes.” He is an assertive type of person. If my work is not that good, he will tell the truth about it which is good for my personal development.”

“I really look up to him and respect him as he is one of the smartest directors in the company. I also trust him because he is open toward discussions and willing to nurture us.”

### Individualized Consideration

Individualized consideration is defined as communicable, helpful, appreciative, and nurturing. Individualized consideration is where he/she is open toward communication and offers support, appreciates his/her subordinates, helps his/her subordinates to grow, and develops their strengths. Leaders with individualized consideration care about their subordinates’ personal development and help their subordinates by having an open communication style and offering support.

#### Employees’ Statements

##### Appreciative

“Although I am a junior in this division, he really appreciates my input and opinions. This type of leader is very positive. For me, when leader appreciates us, it creates motivation.”

“My leader listens to our ideas, is open toward communication and nurtures us, it makes me feel appreciated and motivated.”

##### Nurturing

“My leader nurtures me and my team members. He always encourages us to join the training programs being developed by the company, he persuades us to join like it is good for you in the future, something like that. Even my leader is willing to teach me excel skills personally.”

“My current leader could really nurture my talent; she is not the type of leader that just gives me a task to be completed. She sets milestones for me and is involved in my improvement process; she gives advice and feedback to me.”

## Transactional Leadership

A sub-category identified from the implementation of transactional leadership at X hospital was that of contingent reward.

### Contingent Reward

Contingent reward is a reward-based system developed by a company in order to encourage their employees to meet the company’s goals. It is linked with performance; if an employee reaches a certain target or score, he or she will get bonus and/or benefits. The contingent reward system at X hospital is called the Key Performance Index (KPI). Employees’ annual performance is determined by the score on the KPI; the higher the score, the larger bonus, and benefits they receive. This is not directly implemented by subordinates’ immediate leaders, but is a system instigated by the highest levels of management in the company.

#### Employees’ Statements

“We have performance toward reward linkage in our company, but it is not being implemented by our leader directly; it is the company’s reward system. In every job, there is Key Performance Index which reflects our performance, so performance toward reward linkage exists in our company.”

“Yes, we have performance toward reward linkage in this company. We have set goals on the Key Performance Index, if we achieve this score, we will receive this much amount bonus as a reward for our performance.”

## Democratic Leadership

A sub-category identified from Democratic Leadership at X hospital was Democracy.

### Democracy

There are four attributes of democracy in democratic leadership. Firstly, democratic leaders are open toward decision making and are team-oriented. The second, is that they are open to their subordinates’ advice. Third, they help give job related information and provide all the information needed to accomplish a job’s tasks to their subordinates Lastly, they mentor their subordinates, explaining and telling them how to do the job effectively based on their past experiences.

#### Employees’ Statements

##### Open Toward Decision Making

“He is a type of a democratic leader and not a dominant one; every decision he makes will be passed through us and his peers. For example, there is this one time where my friend, me, and him are in the decision-making process. Me and my friends chose A and he chose B, since we voted for A, A is chosen as the final decision.”

“We can give input on the decision-making process like I have mentioned before, this division has lots of brainstorming sessions, where we discuss and give input on decision making and problem solving.”

## The Leader-Member Exchange Relationships

The Leader-Member Exchange (LMX) relationships in X hospital were identified through interviews with ten employees working there. There was a total of seven questions in LMX 7. The LMX relationships showed that different types of leadership styles result in different answers from the questions of LMX 7. The dyadic relationships between leaders and employees were determined by the type of leadership style implemented by leaders. The results are divided into two sections: the LMX relationships between leaders and employees under transformational and democratic leaders. Transactional leaders that have not been included in this section as “Key Performance Index,” is common in many industries, and interviewees mentioned that the KPI is a reward system that was developed by the company, not directly by their leaders.

Employees’ statements under transformational and democratic leaders:

1. Leader understands his or her job problems and needs.

“Of course, since I am new in this division, he makes sure that we know everything that we need in this work. So, in the first week, it’s like an orientation where he sets up a schedule for what we need to learn on Monday to Friday, like from A to Z since I am new in this division such as learning about health technology, design, and new application and others. He has prepared it since the beginning, and if something misses out, he will explain it to me directly, he will not appoint others to explain it, he will explain it directly.”

2. Leader recognizes his or her potential.

“Since my leader appointed me for a huge project, I think that he recognizes my potential since he had heard of my career from others before in another division in which I am doing good at it, so he appointed me for it. Like the other head of division talked about me and he acknowledged it. Since I made a feature for the mobile apps which was quite popular among heads of divisions before.”
“Yes, when I was a trainee in this company, he told me, I like the way you work and your performance. Just be in this division in your last placement, something like that.
It shows that he sees potential in me and wants me to be in his division which makes me feel appreciated and respected.”

2. Leader uses his or her power to bail him or her out.

“I presented the wrong data, and he took responsibility as my head of division. He said that it’s our fault, not only my fault, although that it was totally my fault.”

3. Working relationship.

“Hmmm, I would say it’s the maximum, so it is going to be extremely effective…he is the best leader that I have met.”

“I would give the maximum for working relationship between me and my boss, extremely effective. It seems like I really appraise him.”
“I would say extremely effective, since she is a kind and supportive person; I really like that about her.”

The working relationship between employees and leaders under transformational and democratic leadership reveals that they tend to have better than average and extremely effective relationships. Furthermore, employees under transformational and democratic leadership answered the seven questions of LMX 7 positively, inferring that they have high-quality leader-member exchange relationships.

### Working Relationship

“Average.”

## Conclusion

The results indicate that of the different leadership styles enacted within X hospital, employees under transformational and democratic leadership styles have better than average to extremely effective working relationships, and a high to a very high quality of leader-member exchange relationships. Employees working under transactional leaders have moderated only to the high quality of leader-member exchange relationships. This study shows that, to have the most effective working relationships, transformational and democratic leadership should be implemented as it leads to a higher quality of leader-member exchange relationships, whereas transactional leadership leads to less effective levels of working relationships, as well as lower quality of leader-member exchange relationships. In the healthcare industry at a time of crisis, a leadership that cares, nurtures, and protects employees is the most effective and productive.

## Author Contributions

MR contributed to conceptualization, methodology, investigation, curation, analysis, funding acquisition, and writing. NA and HA helped in the investigation, curation, analysis, and writing. AP helped in review, analysis, and writing. All authors contributed to the article and approved the submitted version.

## Conflict of Interest

The authors declare that the research was conducted in the absence of any commercial or financial relationships that could be construed as a potential conflict of interest.

## Publisher’s Note

All claims expressed in this article are solely those of the authors and do not necessarily represent those of their affiliated organizations, or those of the publisher, the editors and the reviewers. Any product that may be evaluated in this article, or claim that may be made by its manufacturer, is not guaranteed or endorsed by the publisher.

## References

[B1] Baxter P. Jack S. (2008). Qualitative case study methodology: study design and implementation for novice researchers. *Qual. Rep.* 13 544–559. 10.46743/2160-3715/2008.1573

[B2] BBC (2021). *The death of Indonesian health workers due to Covid-19 is the highest in Asia, what is the price we have to pay in the future?* Jakarta: BBC News Indonesia.

[B3] Chirico F. Nucera G. Magnavita N. (2020). COVID-19: Protecting healthcare workers is a priority. *Infect. Cont. Hosp. Epidemiol.* 41 1117–1117. 10.1017/ice.2020.148 PMC7198459 32299519

[B4] Faculty of Medicine, University of Indonesia (2020). *83% of Indonesian Health Workers Experienced Moderate and Severe Burnout Syndrome During the COVID-*19 *Pandemic.* Depok City: University of Indonesia.

[B5] Fahlevi M. Alharbi N. S. (2021a). Adoption of e-payment system to support health social security agency. *Int. J. Data Network Sci.* 5 737–744. 10.5267/j.ijdns.2021.7.007

[B6] Fahlevi M. Alharbi N. S. (2021b). “The Used of Technology to Improve Health Social Security Agency Services in Indonesia,” in *3rd International Conference on Cybernetics and Intelligent Systems, ICORIS 2021*, Piscataway: IEEE. 10.1109/ICORIS52787.2021.9649649

[B7] Kaye A. D. Okeagu C. N. Pham A. D. Silva R. A. Hurley J. J. Arron B. L. (2021). Economic impact of COVID-19 pandemic on healthcare facilities and systems: International perspectives. *Best Pract. Res. Clin. Anaesthesiol.* 35 293–306. 10.1016/j.bpa.2020.11.009 34511220 PMC7670225

[B8] Purwanto A. Supono J. Rahayu P. Ponda H. Fatma N. F. Fahlevi M. (2020). Develop risk and assesment procedure for anticipating covid-19 in food industries. *J. Crit. Rev.* 7 1991–2004. 10.31838/jcr.07.15.268

[B9] Purwanto H. Fauzi M. Wijayanti R. Al Awwaly K. U. Jayanto I. Purwanto A. (2020). Developing model of halal food purchase intention among indonesian non-muslim consumers: an explanatory sequential mixed methods research. *Syst. Rev. Pharm.* 11 396–407.

[B10] Saunders M. Lewis P. Thornhill A. (2009). *Research Methods for Business Students* (5th ed.). Hoboken: Prentice Hall.

[B11] Sekaran U. Bougie R. (2016). *Research methods for business: A skill building approach.* Hoboken: John Wiley & Sons.

[B12] Sunarsi D. Rohaeni N. Wulansari R. Andriani J. Muslimat A. Rialmi Z. (2020). Effect of e-leadership style, organizational commitment and service quality towards indonesian school performance. *Syst. Rev. Pharm.* 11 472–481. 10.31838/srp.2020.10.71

[B13] Supriadi O. Musthan Z. Nurjehan R. Haryanti Y. D. Rafid Marwal M. Purwanto A. (2020). Did transformational, transactional leadership style and organizational learning influence innovation capabilities of school teachers during covid-19 pandemic? *Syst. Rev. Pharm.* 11:47. 10.31838/srp.2020.9.47

[B14] Vizheh M. Qorbani M. Arzaghi S. M. Muhidin S. Javanmard Z. Esmaeili M. (2020). The mental health of healthcare workers in the COVID-19 pandemic: a systematic review. *J. Diab. Metab. Disord.* 19 1967–1978. 10.1007/s40200-020-00643-9 PMC7586202 33134211

